# Dynamic Neural Fields with Intrinsic Plasticity

**DOI:** 10.3389/fncom.2017.00074

**Published:** 2017-08-31

**Authors:** Claudius Strub, Gregor Schöner, Florentin Wörgötter, Yulia Sandamirskaya

**Affiliations:** ^1^Autonomous Robotics Lab, Institut für Neuroinformatik, Ruhr-Universität Bochum, Germany; ^2^Department of Computational Neuroscience, III Physics Institute, Georg-August-Universität Göttingen, Germany; ^3^Institute of Neuroinformatics, University of Zurich and ETH Zurich Zurich, Switzerland

**Keywords:** dynamic neural fields, intrinsic plasticity, adaptation, dynamics

## Abstract

Dynamic neural fields (DNFs) are dynamical systems models that approximate the activity of large, homogeneous, and recurrently connected neural networks based on a mean field approach. Within dynamic field theory, the DNFs have been used as building blocks in architectures to model sensorimotor embedding of cognitive processes. Typically, the parameters of a DNF in an architecture are manually tuned in order to achieve a specific dynamic behavior (e.g., decision making, selection, or working memory) for a given input pattern. This manual parameters search requires expert knowledge and time to find and verify a suited set of parameters. The DNF parametrization may be particular challenging if the input distribution is not known in advance, e.g., when processing sensory information. In this paper, we propose the autonomous adaptation of the DNF resting level and gain by a learning mechanism of intrinsic plasticity (IP). To enable this adaptation, an input and output measure for the DNF are introduced, together with a hyper parameter to define the desired output distribution. The online adaptation by IP gives the possibility to pre-define the DNF output statistics without knowledge of the input distribution and thus, also to compensate for changes in it. The capabilities and limitations of this approach are evaluated in a number of experiments.

## 1. Introduction

A Dynamic Neural Field is a description of activity of a large homogeneous neuronal population (Wilson and Cowan, 1973; Amari, 1977; Coombes et al., 2014; Schöner and Spencer, 2015). The DNF equation is obtained as a mean-field approximation of the dynamics of a network of spiking neurons and describes the dynamics of a continuous *activation* function, spanned over a *feature dimension*, such as color, location, velocity, or other perceptual or motor parameters, to which the neurons in the underlying population respond.

The core elements of the DNF dynamics are a winner-takes-all type of connectivity, expressed by a symmetrical interaction kernel with a short-range excitation and a long-range inhibition, and a sigmoidal non-linearity. The sigmoidal non-linearity determines the *output* of the DNF. The DNF's output is a function over the feature dimension that vanishes for activation values below zero and saturates at one for positive activation values. The recurrent connectivity pattern and sigmoid output function of the DNF lead to non-linear properties of this model. These properties enabled its successful application in modeling cognitive functions in humans: e.g., formation of a representation, working memory, decision making, rule learning, or executive control (Schöner and Spencer, 2015), as well as for control of cognitive robots (Bicho et al., 2011; Sandamirskaya et al., 2013).

One of the obstacles to a wider adoption of the DNF model in technical systems and in neurobehavioral modeling is the parameter tuning required to obtain the desired behavior. In general, the behavior of a DNF for a given input depends on the parameters of the neural field, e.g., the strength and width of the interaction kernel, the resting level, or the slope of the sigmoidal non-linearity. However, when considering the DNF output behavior over time for sequences of inputs, the particular *input distribution* has a major impact on the DNF output statistics. Therefore, the input distribution has to be taken into account when setting parameters of a DNF in order to achieve a desired behavior over time. When the input distribution is not known in advance and its online normalization is not straight-forward, the tuning of the DNF parameters may be time consuming, in paritular when the input distribution varies (e.g., drifts) over time.

Let us consider an example, which we will use throughout this paper: a robotic hand with a tactile sensor on its fingertip is used to estimate the shape of objects by rotating them and bringing the fingertip in contact with the object in different locations. In this example, we use the tactile sensor on the robotic fingertip as a source of input to a DNF (see Strub et al., 2014a,b for the details of the robotic setup and the DNF architecture). When this sensor is brought into contact with an object, the shape of the contact area is characteristic for the properties of the object's surface at the location of the contact. Thus, a low circularity of the contact area on a tactile sensor (down to zero circularity for a sharp line) corresponds to an edge on the object's surface, whereas high circularity (up to 1 for a perfect circle) corresponds to a flat surface under the sensor. The task we will consider is to create a “map” of flat surfaces of an object (in an object-centered coordinate frame), as the robotic hand rotates the object, repeatedly bringing the tactile sensor on its fingertips in contact with the object at different locations. To build such “map,” we need to detect the most circular contacts within a sequence of contacts and store their position on the object's map. Here, the angular coordinate anchored in the object's center is the feature dimension we are interested in (on which we build a “map” of the detected flat surfaces, using the DNF). The circularity of the contact point determines the activation level of the DNF, induced by the sensory input. The desired output of the DNF in this case is an activity peak for a given fraction of the inputs (e.g., within 20% of the most circular inputs), and no output activity for inputs with lower circularity values.

**Figure 1:**
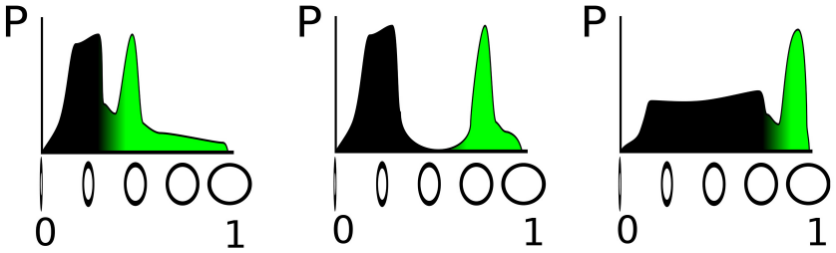
Illustration of three different distributions of a “circularity feature” obtained from sensory input. On the vertical axis, the circularity is denoted, which determines the activation level of a DNF; the horizontal axis shows the probability to measure the respective circularity value. The green filling represents a fixed fraction (20%) of the total probability density.

The parametrization of such a detector depends crucially on the distribution of the circularity feature in the input stream from the sensor, as illustrated in Figure 1. In the figure, the sensed property—circularity in our example, —which determines activation level of the DNF, is plotted on the horizontal axis and its probability of occurrence on the vertical axis, thus showing the distribution of input amplitudes that can be measured. The green-colored part of the distribution corresponds to a fixed fraction of the input distribution, say 20% that includes the highest input circularities. The three examples, shown in Figure 1 illustrate that the classification threshold for the activation of a DNF—which is determined by the negative resting level and the threshold parameter of the sigmoid of the DNF—depends on the particular feature distribution. If the input distribution is not known in advance, or varies over time, an online adaptation of the detection threshold (i.e., resting level) and steepness of the classification function (i.e., steepness of the sigmoid) is necessary.

In this paper, we propose a method to autonomously adapt parameters of a DNF—in particular, the gain of the sigmoid non-linearity and the resting level (bias)—using a homeostatic process. In order to achieve this adaptation, global input and output measures for the DNF have to be defined. Here we use as an output measure the maximum level of the output of the field (where output is the activation after it is passed through the sigmoid function). The corresponding input measure is the activation value of the input at the location of the maximum output. The underlying notion is that the maximum level of the DNF output reflects the decision that the field has made about its input. That decision was based on the input at the selected location. Based on these measures, the gain and resting level for the DNF are adapted in order to match the distribution of this output measure obtained over time to a predefined target distribution. This adaptation drives the DNF dynamics toward the detection instability, which separates the inactive, subthreshold states of the DNF from the active states with a local activation peak. As a result, the DNF is kept in a dynamical regime in which it remains sensitive to input, preventing both saturation and the complete absence of activity. Furthermore, the adaptation ensures that the distribution of the output measure of the DNF remains invariant when the input distribution changes over time, for example, in terms of its mean or variance.

In the following sections, the DNF and IP equations are introduced, the derivation of DNFs with IP is outlined, and the performance of the modified DNF is evaluated on an example in which input from a tactile sensor is processed.

## 2. Methods

### 2.1. Dynamic neural fields

DNFs are dynamical systems which model activation dynamics in large homogeneously connected recurrent neuronal networks. The DNF equation describes an activation function that may represent a perceptual feature, location in space, or a motor control variable (Schöner and Spencer, 2015). This behavioral variable is encoded along a feature dimension *x* of the DNF, and the activation *u*(*x, t*) at position *x* encodes the confidence that the feature has value *x* at time *t*. The current state of the neuronal system is encoded by the position on the dimension *x* of high activation values. Such space coding allows to encode multiple possible values of a feature as well as “fuzziness” of the experienced or stored values. Other artificial neural networks encode feature values through the level of activity of particular neuronal units (rate coding) or through the pattern of activation across a distributed set of units. What is special about the space code used in DNF is that the metric distance between represented values is explicitly encoded in the distance between locations along the feature dimensions. Neural coupling that depends on the distance between field locations thus depends similarly on the distance between the represented feature values.

The equation for the DNFs used in the proposed model is described by Equation (1), which defines the rate of change in activation *u*(*x, t*) of the field:

(1) $$\begin{array}{l} \tau \dot{u}(x,t)=-u(x,t)+h+S(x,t)\\ \;+\int \omega (|x-x\prime |)g(u(x\prime ,t))dx\prime . \end{array}$$

In Equation (1), *u*(*x, t*) is the activation of the DNF at time step *t* and position *x*. The position *x* describes a feature dimension and may be multi-dimensional: $\vec{x}\in {\mathbb{R}}^{n}$. In practice, the dimensionality of fields ranges from zero (neural nodes) to three or four. In this paper only one dimensional fields are considered.

The term −*u*(*x, t*) stabilizes an attractor for the activation function at values that are defined by the last three terms in the equation. The time constant, τ, determines how fast the activation pattern, *u*(*x, t*), relaxes to the attractor. The negative resting level, *h*, ensures that the DNF produces no output in the absence of external input, *S*(*x, t*). The convolution term models recurrent neural interactions between activation levels at different locations within the DNF, and is shaped by the interaction kernel:

(2) $$\begin{array}{l} \omega \left(\left|x-x^{\prime }\right|\right)=c_{exc}\exp\left[-\frac{{\left(x-x^{\prime }\right)}^{2}}{2\sigma_{exc}^{2}}\right]-c_{inh}\exp\left[-\frac{{\left(x-x^{\prime }\right)}^{2}}{2\sigma_{inh}^{2}}\right] \end{array}$$

with a short-range excitation (strength *c*_*exc*_, width σ_*exc*_) and a long-range inhibition (strength *c*_*inh*_, width σ_*inh*_ > σ_*exc*_). A sigmoidal non-linearity, *g*(*u*(*x, t*)) = (1 + exp[−β*u*(*x, t*)])^−1^ defines the output of the DNF through which the DNF impacts on other neural dynamics within a neural architecture, and also on its own neural dynamics through the recurrent interactions.

**Figure 2:**
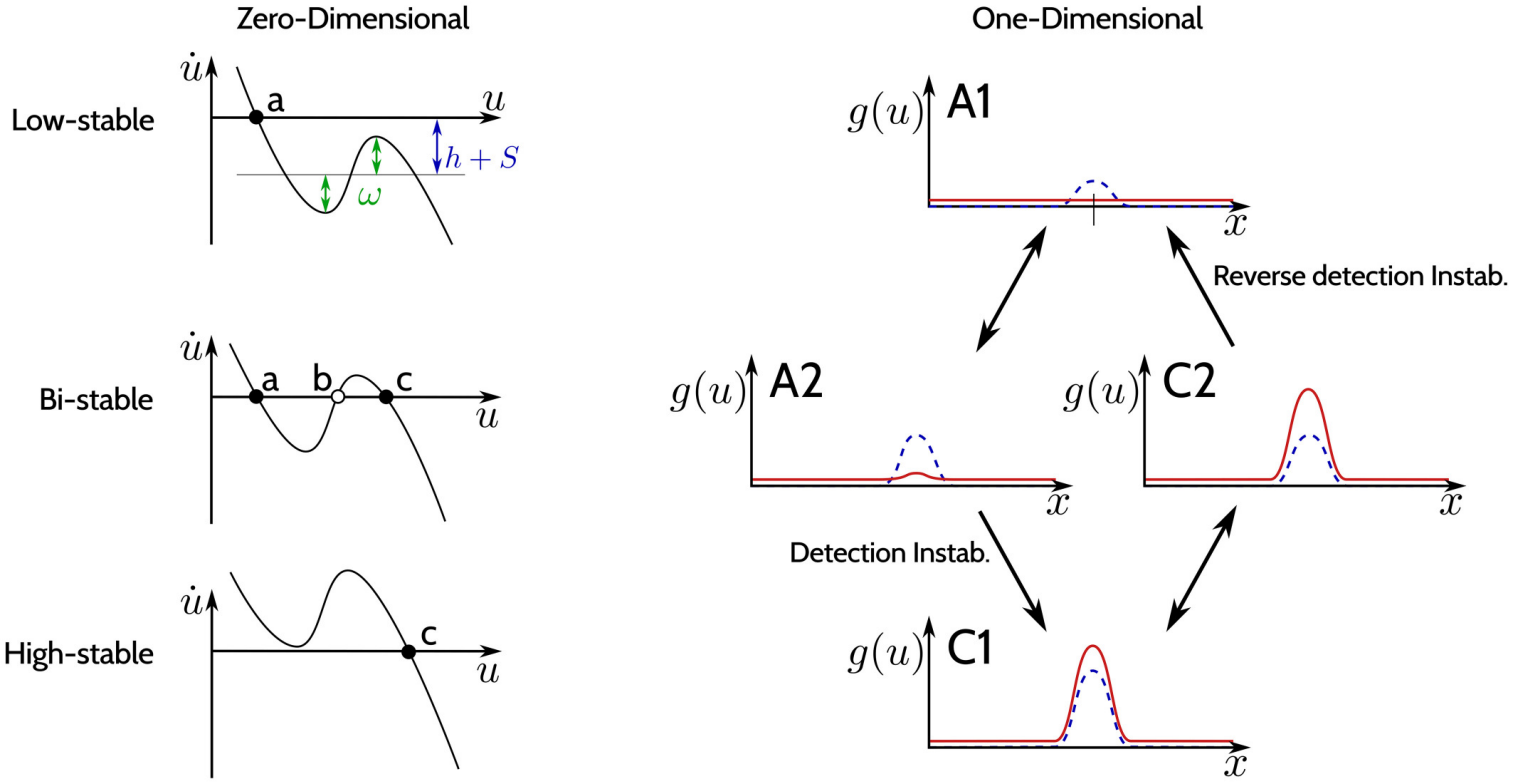
The three regimes of stability. (**Left column**): Phase plots for different regimes of the DNF equation for a zero-dimensional feature space *x* (*u* is a scalar value). Black dots indicate stable fixed points, empty circles—unstable fixed points. (**Right column**): The output *g*(*u*) of a DNF is illustrated (in red) for an one-dimensional feature space *x*. The blue-dashed line represents the input *S*(*x*). The arrows depict qualitative changes in the regimes of stability determined by the input strength *S*(*x*).

The −*u*(*x, t*) in Equation (1) guarantees the existence of at least one attractor. Dependent on the parametrization of the recurrent interaction kernel ω and the sum of the input signal *S* and the resting level *h*, the DNF may undergo saddle-node bifurcations. Figure 2 shows schematically the bifurcations that a DNF undergoes when the sum *h*+*S*(*x*) changes. The left column of the figure shows a zero-dimensional case (when *u* is a scalar value and the state is a point), and the right column shows a one-dimensional case [when *u*(*x*) is a function and the state corresponds to a line].

The phase plots on the left in Figure 2 qualitatively show Equation (1) for a zero-dimensional state *x* (i.e., a point) at different input values:

In the top plot (“Low-stable”), the black dot denotes a single stable fixed point (attractor) (a) for the case when *h*+*S* is below activation threshold of the DNF. Loosely speaking, the resting level *h* together with the input *S* of the DNF shift the function of the phase plot up and down, while ω determines the non-linearity of the function which general shape is determined by the sigmoid *g*. A stronger input intensity *S* may cause a bifurcation, creating two new fixed points: a stable (c) and an unstable one (b) (“Bi-stable” regime, middle plot). If the input further increases, a second bifurcation occurs, where the unstable fixed point (b) collides with the stable fixed point (a) in the phase plot. Now the system state at the former fixed point (a) has lost its stability and the system will converge to the remaining stable fixed point (c) (“High-stable,” lower plot). This second bifurcation, where the bi-stable state looses stability and switches to the high-stable state is termed *detection instability*. If the input *S* now decreases again, it will induce a bifurcation, leading back to the bi-stable regime, however the system will remain at the stable fixed point (c) (hysteresis). Only if the input decreases enough to induce the second bifurcation, where the stable fixed point (c) collides with the unstable fixed point (b), the system will return to the stable fixed point (a) (“Low-stable”).

In the one-dimensional system in the right column of Figure 2, the output activation *g*(*x*) over the feature space *x* is plotted in red while the input *S*(*x*) is plotted as a dashed blue line. In the “Low-stable” case, when the sum *h*+*S*(*x*) does not reach activation threshold anywhere in the dimension *x*, the output of the DNF is zero (top plot A1). If the input intensity increases, the system enters a “Bi-stable” regime (middle plot A2), with a weak positive output. When input strength further increases, the detection instability leads to a visible change of the DNF output, which has now a localized “peak” that might even surpass the input strength (lower plot, C1). If the input intensity is decreased now, the system will enter the bi-stable regime again (middle plot, C2), however, without a qualitative change in the system output. The system output drops back to the resting level activity in (A1) only if input is further decreased, which is termed the “reverse detection instability.” For high values of lateral excitation, a negative (inhibitory) input is required for the system to return to an inactive state. This parametrization is termed “self-stabilizing,” i.e., maintaining the output in the absence of input *S*(*x, t*) = 0.

Thus, the recurrent interactions by the kernel ω stabilize the system in its state (either “low” or “high”) when the input fluctuates around the bistable setting by shaping the non-linearity in the phase-characteristics of the system.

### 2.2. Intrinsic plasticity

Neurons in biological organisms have a large spectrum of plasticity mechanisms, implementing a broad range of functions. One functional class of neuronal plasticity mechanisms is termed “homeostatic plasticity,” which optimizes the information processing within a neuron by keeping the firing rate of the neuron in a reasonable regime (Turrigiano, 2008, 2012; Pozo and Goda, 2010).

Non-synaptic, i.e., intrinsic forms of homeostatic plasticity are termed “intrinsic homeostatic plasticity” (IP), which adapt the intrinsic excitability of a neuron (Frick and Johnston, 2005; Schulz, 2006). Additionally to the adaptation in the neuron soma, this plasticity of excitability has also been discovered in compartmentalized dendritic structures of neurons (Frick and Johnston, 2005; Losonczy et al., 2008; Makara et al., 2009). The plasticity of excitability of dendritic structures greatly increases the complexity and non-linearity of neural information processing and storage (Branco and Häusser, 2010; Remy et al., 2010; Spruston et al., 2016).

In the context of artificial neural networks IP is modeled as a mechanism which modifies the excitability of a neuron in order to achieve a specified output distribution for a given input distribution (Stemmler and Koch, 1999; Triesch, 2005). This is done by manipulating the parameters of a transfer function, which transforms the internal neural state to an output. A commonly used transfer function is the logistic function, defined in Equation (3):

(3) $$\begin{array}{l} g_{a,b}(x)\;={\left(1+\exp\left(-ax+b\right)\right)}^{-1}. \end{array}$$

The *a, b* are termed gain and bias of the function *g*(*x*) and *x* is the input which is transferred to the output space. By choosing an appropriate gain and bias, the input may be scaled and shifted in order to cause a response in the desired part of the sigmoid function *g*(*x*). The objective of IP is to adjust the gain and bias such that for a given set of inputs *X* the corresponding set of outputs *g*(*X*) approximates a predefined target distribution. Hence, IP is an autonomous adaptation of the sigmoid transfer function.

The particular IP learning rule for adapting the parameters of the transfer function is achieved by minimizing the Kullback-Leibler-divergence (KLD) (Kullback and Leibler, 1951), such that the output distribution of a neuron is close to the target distribution. A common target distribution is the exponential distribution, as it reflects aspects of homeostasis, i.e., maximizing the transmitted information (entropy) while minimizing the positive mean output activity (metabolic costs) defined by the mean of the distribution. For logistic functions (Equation 3) and the exponential as a target distribution the learning rules have been derived in Triesch (2005). The procedure will only be sketched in the following. For neurons using the tanh as transfer function, see Schrauwen et al. (2008).

(4) $$\begin{array}{l} L_{\text{KL}}\left(f_{g}||f_{\exp}\right)=E_{x}\left[L_{\text{KL}}\left(f_{g}(g_{a,b}\left(x\right)\right)||\frac{1}{\mu }\exp\;\left(\frac{-g_{a,b}(x)}{\mu }\right))\right]\\ \;=\int f_{g}(g_{a,b}(x))\log\left(\frac{f_{g}(g_{a,b}(x)}{\frac{1}{\mu }\exp\;\left(\frac{-g_{a,b}(x)}{\mu }\right)}\right)\text{ d}x, \end{array}$$

(5) $$\;f_{g}\left(g_{a,b}(x)\right)=\frac{f_{x}(x)}{\frac{\partial (g_{a,b}(x))}{\partial x}}.$$

In Equation (4) a Loss function *L* is defined as the KLD *L*_*KL*_ with the probability distribution function *f*_*g*_ of the outputs of the logistic transfer function *g*_*a,b*_(*x*), with respect to the exponential target distribution *f*_exp_. The output distribution of the logistic function *g*_*a,b*_(*x*) is parameterized by *a, b*, while the parameter of the exponential distribution is the mean μ. The output distribution *f*_*g*_(*g*_*a,b*_(*x*)) is defined as the input distribution *f*_*x*_(*x*) remapped by the sigmoid *g*(*x*) (Equation 5). Minimizing the KLD is done by taking the derivative with respect to (*a, b*) and performing gradient descent with a learning rate of η (the step size). This leads to the learning rules for adaptation of the parameters gain *a* and bias *b* with the learning rate η (Triesch, 2005):

(6) $$\begin{array}{l} \Delta b=\eta \left(1-\left(2+\frac{1}{\mu }\right)g_{a,b}(x)+\frac{1}{\mu }g_{a,b}{\left(x\right)}^{2}\right), \end{array}$$

(7) $$\begin{array}{l} \Delta a=\frac{\eta }{a}+x\Delta b. \end{array}$$

Besides this online adaptation rule, a batch version of IP was derived in Neumann and Steil (2011). The application of IP has repeatedly been reported to improve performances in reservoir computing—a particular form of computing with transients in dynamical systems—(Steil, 2007a,b; Wardermann and Steil, 2007; Schrauwen et al., 2008) as well as increasing the robustness with respect to the parameter initialization (Neumann and Steil, 2011). It has been noticed that in a network of neurons adapted by IP the target distribution is also approximated on the network level (Steil, 2007a). There have been a number of variations of IP learning with respect to the target distribution, for further information see (Verstraeten et al., 2007; Schrauwen et al., 2008; Boedecker et al., 2009a,b). Furthermore, combinations of IP with other forms of plasticity have been investigated, e.g., with Hebbian learning, which leads to identification of independent components in the input (Triesch, 2007).

Finally, it should be noted that IP leads to instability of recurrent neural networks (RNN). In Marković and Gros (2010, 2012) the authors show that introduction of IP in RNN leads to the destruction of the attractor stability, resulting in spontaneous and continuously ongoing activity for networks without and with very small input amplitudes. The result of RNN destabilization by IP has also been confirmed in spiking neural networks (Lazar et al., 2007). These destabilizing effects on the dynamics are relevant for applying IP in dynamic neural fields as will be discussed in this paper.

The adaptation of the intrinsic plasticity via stochastic gradient descent can be optimized by utilizing the concept of a natural gradient, introduced in Amari (1998). It has been shown, that the metric structure of the parametric space of neural networks has a Riemannian character (Amari, 1998). Thus the relationship between the distance of two sets of parameters and the distance in the output space of the transfer function is non-linear. Adapting the conventional gradient with respect to the Riemannian metric corrects for this non-linearity, such that the distance of two parameter sets linearly transfers to the output space. This change of the gradient is termed *natural gradient* and leads to a substantial performance increase in the convergence rate for IP (Neumann and Steil, 2012; Neumann et al., 2013). Therefore the natural gradient is used in this paper due to these technical benefits, although the adaptation of DNFs with IP proposed in this paper in principal also works with the standard IP adaptation.

A natural gradient-based parameter adaptation for IP termed NIP has been derived in Neumann and Steil (2012), here only the resulting learning rules are given:

(8) $$\begin{array}{l} \Delta \vec{\theta }=-\eta {\left(F(\vec{\theta })+\epsilon I\right)}^{-1}\nabla_{E}L_{\text{KL}}\left(f_{g}||f_{\exp}\right)\\ \;=-\eta \nabla_{F}L_{\text{KL}}(f_{g}||f_{\exp}), \end{array}$$

(9) $$F(\vec{\theta })=E_{x}\left[\nabla_{E}L_{\text{KL}}(f_{g}||f_{\exp})\cdot \nabla_{E}^{T}L_{\text{KL}}(f_{g}||f_{\exp})\right].$$

The standard gradient of the loss function in an Euclidean metric ∇_*E*_ is transformed into a gradient in the Riemannian metric ∇_*F*_ by inverting the Matrix $F\left(\vec{\theta }\right)$, which is the Fisher information, i.e., the Riemannian metric tensor. In order to prevent numerical instabilities of the tensor inversion, a Tikhonov regularization is applied by adding the identity matrix *I* with a small regularization factor ϵ to the tensor $F\left(\vec{\theta }\right)$ before inversion. Just as before, the loss function *L*_*KL*_ is the KLD for neuron output *g*_θ_(*x*) and parameters *a, b*. As the needed expectation value of the gradient with respect to the input in Equation (9) is not available in an online framework, the tensor $F\left(\vec{\theta }\right)$ is estimated online by:

(10) $${\hat{F}}_{t+1}(\vec{\theta })=(1-\lambda ){\hat{F}}_{t}(\vec{\theta })+\lambda \nabla_{E}L_{\text{KL}}(f_{g}||f_{\exp})\;\cdot \;\nabla_{E}^{T}L_{\text{KL}}(f_{g}||f_{\exp}).$$

with λ realizing a low pass filter with exponential decay which is set to 0.01. For computational efficacy the inversion of the tensor *F* in every time step (Equation 8) can be circumvented by directly estimating the inverse tensor *F*^−1^ as described in Park et al. (2000). Using NIP gives a good approximation of the gradient direction in parameter space, which is also confirmed by the experiments in the evaluation section.

## 3. Intrinsic plasticity for dynamic neural fields

Intrinsic plasticity (IP) is a local adaptative mechanism that models the autonomous adaptation of the sensitivity (gain) and threshold (bias) of a single neuron in order to match the statistics of the neuron's output to a predefined target distribution. We apply this idea to DNFs with respect to a global gain and a global bias parameter that control the entire population of neurons in a DNF. DNFs are a mean field approximations of homogeneous recurrent networks to capture the qualitative, global patterns. Our proposed application of IP on a population level directly tunes the DNF output distribution and therefore achieves the same effect (on the network level) as IP in single neuron would. Thus, conceptually IP in DNFs captures the qualitative, global pattern change in a network as would result form IP in every single neuron.

DNFs are consistent with population coding, in which the value of a feature is encoded by the activity of those neurons within a population that are broadly tuned to that value. If particular feature values never occur in the input, the corresponding neurons never become active. Adapting the gain and bias of each neuron individually would lead to each neuron approximating the desired target distribution. The output of the population would converge to an uniform distribution of feature values, reducing the effectiveness of population coding. The adaptation of a *global* gain and bias for all neurons in a population of a DNF proposed here ensures that the encoding of the input in the DNF activity remains stable. In the Discussion we briefly review evidence from computational neuroscience that supports this notion of global adaptation.

To implement IP in a DNF, the field equation needs to be slightly reformulated. The standard formula of a DNF is given in Equation (1) where the logistic transfer function *g*(·) is now used in the parametric version:

(11) $$\begin{array}{l} g_{a,b}(x)={\left(1+\exp\left(-ax+b\right)\right)}^{-1}. \end{array}$$

The gain, *a*, controls the steepness of the sigmoid and the bias parameter, *b*, controls the position of the zero-crossing of the sigmoid. The bias defines a gain dependent resting level, *b* = *ah*, which replaces the former static resting level, *h*, in Equation (1). The gain, *a*, scales all weights, i.e., is a scaling factor of the input, *S*(*x, t*) together with the recurrent, lateral interaction kernel, ω(*x, x*′), in Equation (1). As all the weights are jointly scaled, the relative contributions of input signal and lateral interaction remain fixed.

Furthermore, three design choices have to be made for deriving the IP learning rules:

1. Define a scalar measure, *z*, of the input of the field.
2. Define a scalar measure, *y*, of the output of the field.
3. Chose the desired target output distribution.

Concerning the first two points, the *output measure*, *y*(*t*), of the field is defined as the maximum output of the neural field

(12) $$\begin{array}{l} y(t)=\underset{x}{\max}\left(g_{a,b}(u(x,t))\right). \end{array}$$

The *input measure*, *z*(*t*), of the field is given by the field activation at the position of the maximum output

(13) $$\begin{array}{l} z(t)=u\left(\underset{x}{\text{argmax}}\left[g_{a,b}\left(u(x,t)\right)\right],t\right). \end{array}$$

Hence, the input for IP is a composition of the actual field input and lateral field interactions, reflecting recurrent components of the neural dynamics. The main advantage of this measure is that it does not alter the output range. If the field output activity is in the range of (0, 1), for instance, the max(·) is in that range too. This removes the need for an additional processing step of input normalization and parameter tuning.

Two alternative definitions would be the integrated (i.e., summed) or the mean of the field output activity. In contrast to the maximum, these are sensitive to the particular parametrization of the recurrent lateral interaction kernel (i.e., the peak size) with respect to the DNF size. Hence, both of these alternative measures require a tuning of the target distribution parameters with respect to the particular DNF parametrization and are therefore neglected. Moreover, choosing the integrated output activity of a DNF as field output would make the output distribution more sensitive to the simultaneous occurrence of multiple peaks.

The *target output distribution* of *y* is set to the exponential distribution with mean μ, implying a sparseness constraint on the field output with respect to the output over time:

(14) $$\begin{array}{l} T\left(y\left(t\right)\right)=\frac{1}{\mu }e^{-\frac{y\left(t\right)}{\mu }}. \end{array}$$

The exponential distribution is particularly suited when the DNF output is desired to be near zero for the majority of inputs (i.e., most of the time) and output activity is only required for a minority of the inputs. Furthermore the exponential is the maximum entropy probability distribution for a specified mean which is optimal with respect to the information transfer. Thus, an exponentially distributed DNF output corresponds to an optimization of the information encoding in the DNF which remains stable during changes in the input statistics, e.g., mean or variance.

With these design choices, the optimization problem is equivalent to the one in Triesch (2005) (described in Section 2.2) and the learning rules for adapting the gain *a* and bias *b* are given by:

(15) $$\begin{array}{l} \frac{\Delta a}{\Delta t}=\frac{\eta }{a}+\frac{\Delta b}{\Delta t}z\left(t\right), \end{array}$$

(16) $$\begin{array}{l} \frac{\Delta b}{\Delta t}=\eta \left(1-(2+\frac{1}{\mu })\;y\left(t\right)+\frac{1}{\mu }y{\left(t\right)}^{2}\right). \end{array}$$

The learning rate η is set to 0.001 and μ to 0.2.

**Figure 3:**
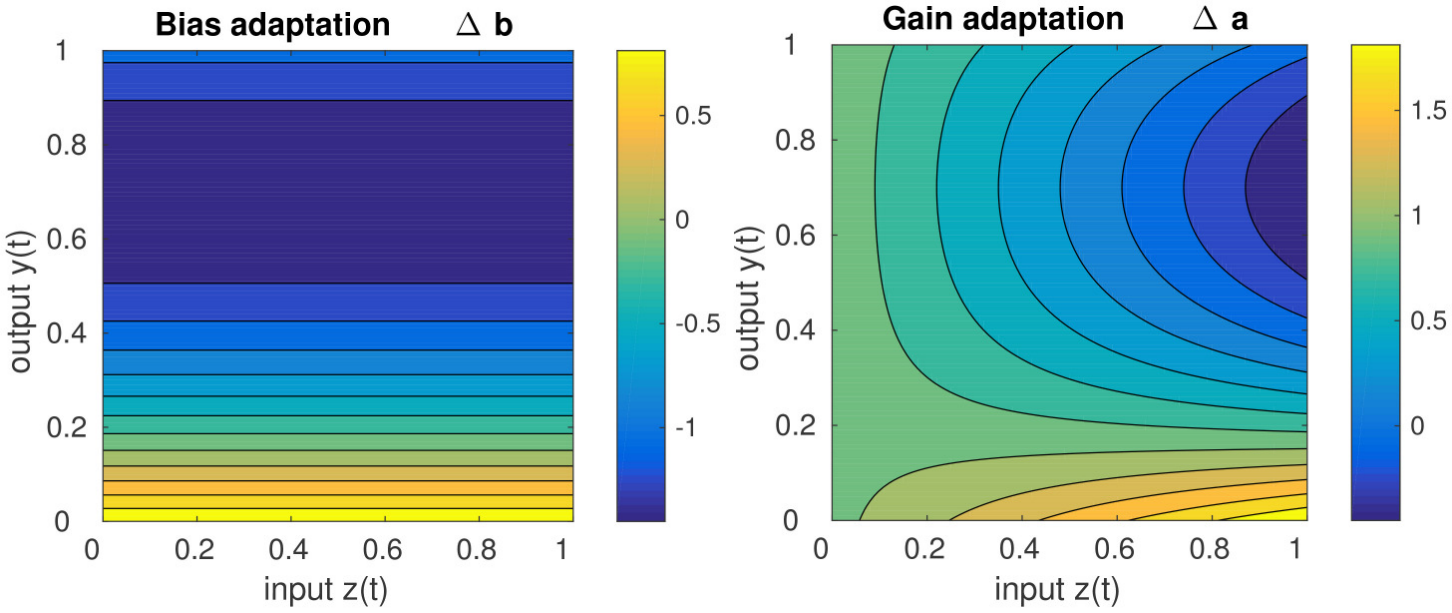
Sketch of the gain adaptation in Equation (10) (**right**) and the bias adaptation in Equation (16) (**left**) for μ = 0.2, input *z*(*t*) in the range of [0, 1], a learning rate η = 1, and a current gain of 1.

Concerning the impact of IP on the stability of the DNF dynamics, it should be noted that IP drives the dynamics toward the detection instability, i.e., to the “edge of stability.” This becomes apparent, when inspecting the behavior of the learning equations Equations (15, 16), depicted in Figure 3. It is visible that for high DNF output, the bias (i.e., resting level) is decreased while for low DNF output the bias is increased, independent of the input. While the gain adaptation depends on the bias adaptation and the current input, the principal “direction” of adaptation is the same as for the bias: high output with high input leads to a decrease of the gain, low output with high input leads to the gain increase. Hence, the parameters are adapted such that IP leads to the destruction of attractor in which the system state currently is. In the long run, this enforces an oscillation between the two attractors [the stable fixed points (a) and (c)] in Figure 2, i.e., drives the system repeatedly through the detection instability. Therefore, IP prevents the DNF from operating in a self-stabilizing regime where the recurrent interaction is sufficient for maintaining the system output, independent of the input. A DNF with IP will operate in a regime where the system state regularly runs through the detection instability—driven by the input.

The parameter adaption of IP can be significantly improved with respect to the convergence speed and robustness by computing the natural gradient (Section 2.2). Therefore, the gradient direction and amplitude of Δ*a* and Δ*b* is corrected by a metric tensor imposing a Riemannian structure in parameter space, i.e., the natural gradient is computed as described in Neumann et al. (2013). The tensor decay parameter λ in Equation (10) is set to $\frac{\tau }{1,000}$ where τ is the time constant of the DNF equation in Equation (1). The regularization parameter ϵ in Equation (8) of the tensor inversion is set to 0.0001.

## 4. Evaluation

**Figure 4:**
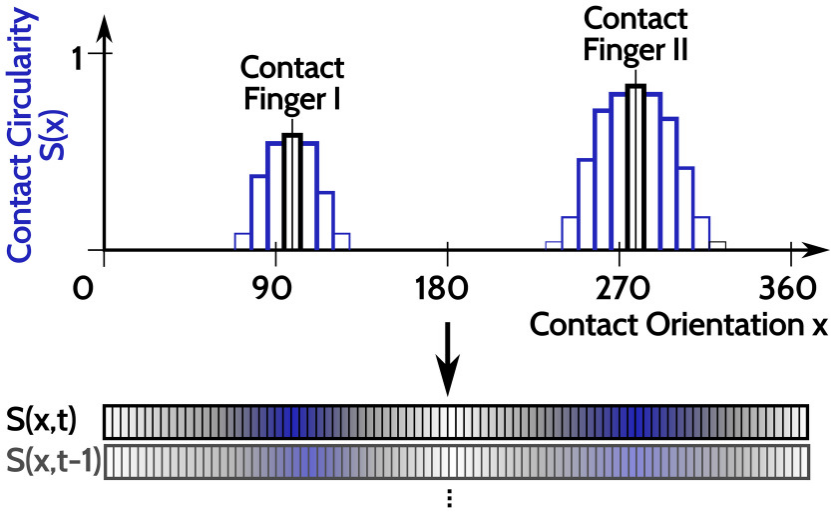
Sketch of the input encoding used for evaluation of DNFs with IP, illustrated for two tactile contacts at opposing orientations (x = 95 deg and x = 275 deg). A population of neurons encode the contact circularity over contact orientation, with each neuron encoding a specific orientation. The corresponding neurons representing the orientations of the tactile inputs are activated and their response strength is related to the contact circularity of the tactile contacts (the two black bars). The Gaussian blurring of the neuronal activation to neighboring neurons (encoding similar orientations) is depicted in the blue bars. This population representation of tactile inputs is done for every time step, leading to the input time series *S*(*x, t*).

For evaluating the DNF with IP, an input time series is constructed from haptic recordings of robotic object manipulations done in Strub et al. (2014a). Two features were used from these data, the orientation of a contact ([0, 360deg]) and its spatial shape on the two dimensional tactile sensor arrays. The spatial shape of the contact is rated between (0, 1), dependent on how “circular” its shape is: 1 corresponds to a perfect circular contact shape and 0 corresponds to a sharp line on the tactile sensor. As the object manipulations are done with two fingers, there may be none, one, or two simultaneous contacts at every point in time (originating from both fingers but with opposing contact orientations). A one dimensional population code is generated from these two features, as depicted in Figure 4. A population of neurons encodes the contact shape over the contact orientation, where the output rate of each neuron (bars in Figure 4) signals the confidence that there is a circular contact at the orientation which the neuron encodes (position along the x axis in Figure 4). The neuron response is blurred with a Gaussian filter across the contact orientation dimension, depicted by the blue bars in Figure 4. This population encoding of the tactile input is accordingly done for every step in time and the resulting time series of the recorded dataset is looped in order to present it for arbitrarily long periods.

With this setup the following cases are evaluated (an average one and three limit cases):

1. Input with average amplitudes [0, 6] for μ = 0.1 and μ = 0.2;
2. Input with low amplitudes [0, 1] (i.e., ÷6);
3. Input with high amplitudes [0, 36] (i.e., ×6);
4. Input with high offsets [−12, −6] (i.e., −12) for IP with and without natural gradient.

These limit cases were selected, since they are quite common in situations when DNFs are driven with sensory inputs and lead to incorrect behavior: high amplitude input might saturate the field, whereas low amplitude might render the field unresponsive. Both effects can occur if input distribution is scaled or shifted. The goal in all these experiments is to detect the most circular contacts with the DNF, i.e., the DNF output should give a peak if the relative input “circularity” is sufficient to be classified as a circular contact and have zero output otherwise. This classification into two classes depends on the particular distribution of the circularity feature.

### 4.1. Varying the mean

In the first set of experiments, the input time series is fed into a one-dimensional DNF with IP, for two different means (μ = 0.1 and μ = 0.2) of the target exponential distribution. These values are in the range of biological neurons in the cortex (see e.g., Hromádka et al., 2008; Barth and Poulet, 2012; Margolis et al., 2012). The aim here is to point out the qualitative influence of the target distribution mean on the DNF output.

The recurrent interaction kernel is parametrized with: *c*_*exc*_ = 14, σ_*exc*_ = 2, *c*_*inh*_ = −7, σ_*inh*_ = 6 and the DNF is sampled at 100 points (i.e., a size of [1,100]). The setup is run with presenting the input time series based on recorded data in realtime (3 fps) and the DNF with IP has a τ of 100 ms and is updated with an Euler step width of 10 ms. The DNF with IP is run until the parameter adaptation by IP does not change qualitatively, i.e., it has converged.

**Figure 5:**
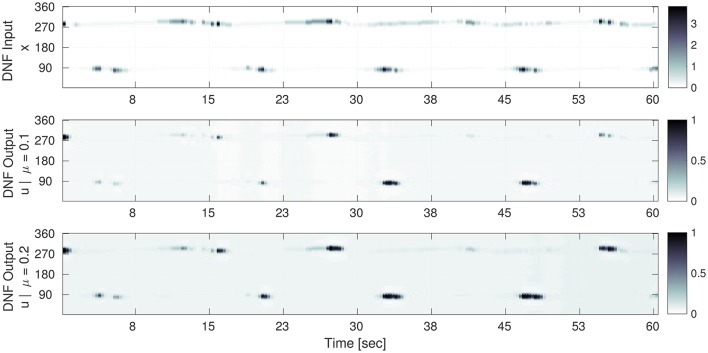
Selection of the input time sequence *S*(*x, t*) and the corresponding DNF output *g*(*u*(*x, t*)) for converged gain and bias adaptation. Time is on the horizontal axis and the one dimensional population code is on the vertical axis. (**Top**) The input time series *S*(*x*) to the DNF. Here, the gray level encodes the input amplitude *S*(*x*) at the corresponding contact orientation x (vertical axis) for a point in time. (**Middle**) (μ = 0.1): the DNF output for the converged IP parameters (*a* = 0.65 and *b* = −3.5). The input-output correlation (Equation 17) for the shown sequence is 0.69. (**Bottom**) The DNF output for the converged IP parameters (*a* = 0.59 and *b* = −3.0) for μ = 0.2. The input-output correlation is 0.67. In the middle and bottom plots, the gray level encodes the DNF output activity *g*(*u*), i.e., surface detection at the corresponding contact orientation x (vertical axis).

A selection of the input sequence and the corresponding output sequence of the DNF in this setup is shown in Figure 5. In the top row the DNF input amplitude (i.e., intensity) *S*(*x*) is shown for contact orientations along the vertical axis of the plot for a given point in time (horizontal axis). The dark regions encode high input amplitudes at the corresponding contact orientation (vertical axis), see the gray-level bars on the right of the figure. The corresponding output of the DNF for the converged IP parameters is shown in the bottom row of Figure 5.

It is noticeable, that the processing by the DNF results in a “sharpened” version of the input, where the structure is preserved. The IP hyper-parameter μ determines how “sensitive” the DNF is with respect to the input: for μ = 0.1 peaks are only generated for the highest input intensities, for a mean of μ = 0.2 the DNF generates more peaks in time which also tend to last longer. The difference in the converged parameters between the two cases is a decrease of the gain *a* of 0.06 (−10%) and an increase of the bias *b* by 0.5 (+15%).

### 4.2. Varying the input distribution

**Figure 6:**
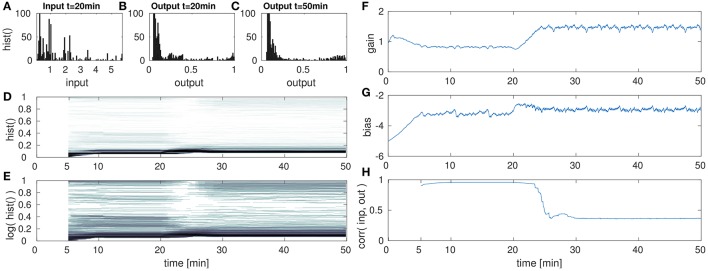
DNF with IP for low amplitude input after the 20th min. **(A)** DNF input histogram, *z*(*t*), **(B)** DNF output histogram, *y*(*t*) after IP parameter convergence at the 20th min. **(C)** DNF output histogram, *y*(*t*) at the 50th min after the input down-scaling. **(D)** DNF output histogram over time, **(E)** logarithmic version of **(D)**, **(F–H)** gain, bias and the input-output correlation over time, respectively. See text for further description.

**Figure 7:**
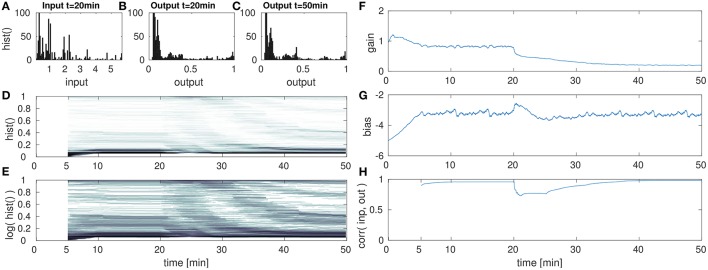
DNF with IP for high amplitude input after the 20th min. **(A)** DNF input histogram *z*(*t*), **(B)** DNF output histogram, *y*(*t*), after IP parameter convergence at the 20th min. **(C)** DNF output histogram at the 50th min after the input up-scaling. **(D)** DNF output histogram over time, **(E)** logarithmic version of **(D)**, **(F–H)** gain, bias and the input-output correlation over time, respectively. See text for further description.

**Figure 8:**
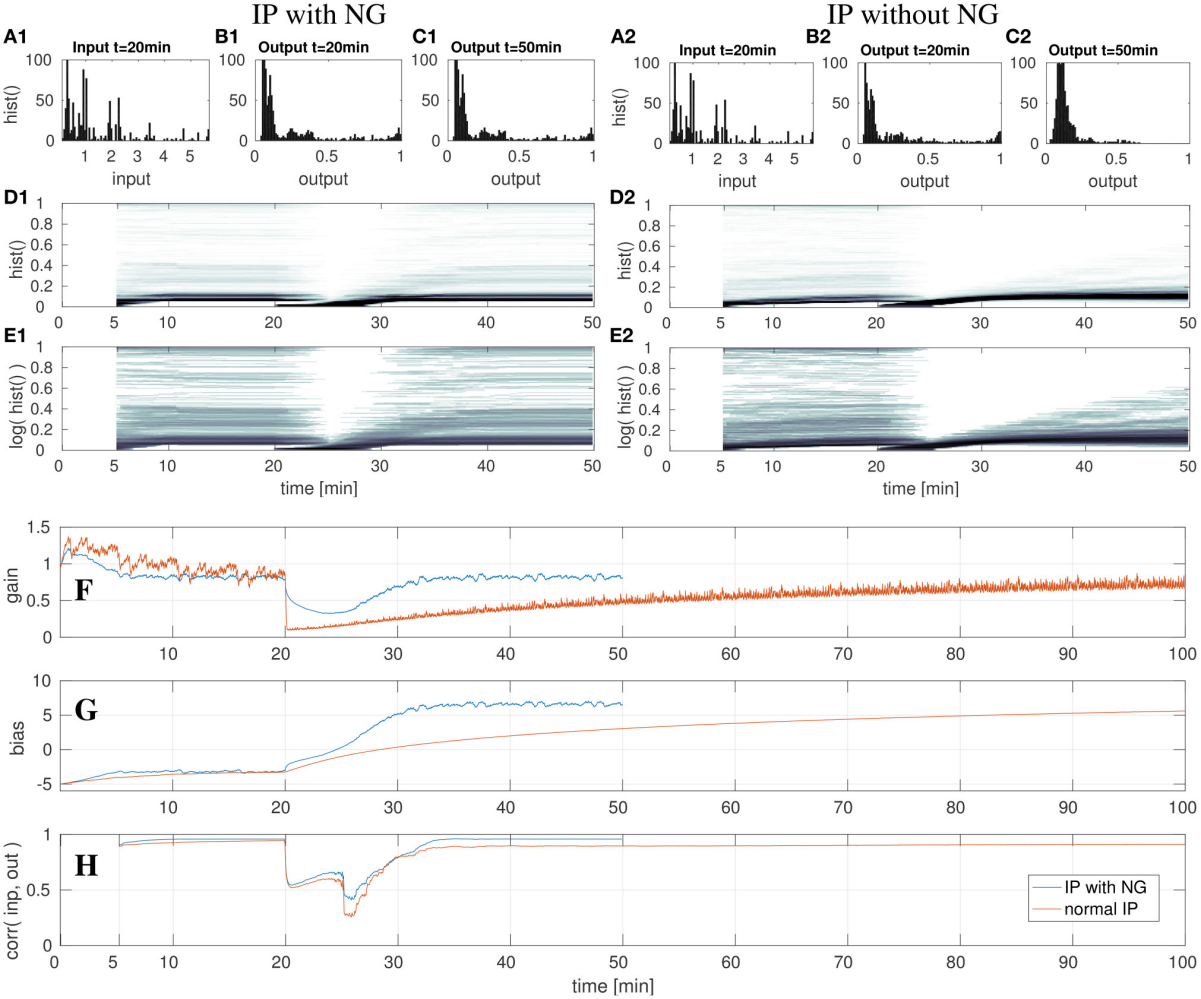
DNF with IP and shifted input after the 20th min with- and without the natural gradient. On the left three top rows show the results for IP with natural gradient descent. The right three rows show the results when using the gradient descent in euclidean parameter space. Shown are the input *z*(*t*) **(A)** and output *y*(*t*) **(B,C)** histograms of the DNF. The output histograms over time **(D,E)** show the output distributions over time, computed by a sliding time window of 5 min. See text for further description. The lowest three rows show parameter adaptation in a DNF with IP and shifted input after the 20th min with- and without the natural gradient. The parameter adaptation is shown for the gain **(F)** and bias **(G)**, and the input-output correlation **(H)** is plotted. The experiment with NG is stopped after the 50th min, the experiment without NG is run until minute 100. See text for further description.

In the following set of experiments the impact of a sudden change in the input distribution is analyzed. This could result e.g., from tactile exploration of a new object with different geometry (i.e., circularity distribution) or changes in the tactile exploration speed or strategy. For this the input sequence is presented for four cycles (i.e., 20 min in the experimental setup) in order to let the IP parameter adaptation converge. After the 20th min, the input is manipulated in its variance (scaled) or its mean (shifted). Then the parameter adaptation by IP is analyzed for the succeeding 30 min. The results of this evaluation are shown in the Figures 6–**8**.

These figures show histograms of the unmodified input *z*(*t*) (Figures 6A, 7A, 8A) as defined in Equation (8) and the output of the DNF *y*(*t*) for the original input (Figures 6B, 7B, 8B) defined in Equation (7). In (Figures 6C, 7C, 8C) the DNF output distribution is shown after learning has adapted the system to the manipulated input statistics and the experiment is stopped (i.e., after 50 min). All histograms are computed within a 5 min time window. Furthermore, in (Figures 6D, 7D, 8D) the output distribution (vertical axis) is plotted over time (horizontal axis) with a 5 min sliding time window to estimate the distribution. The size of the time window was chosen such that it contains one full input period (approximately 5:15 min with 3.33 fps) representative for the input distribution. The color intensity encodes the occurrence of the output value during this time window, where white corresponds to no occurrence and black to 100+ occurrences (similar to the plots Figures 6A–C, 7A–C, 8A–C). For an enhanced visualization of the output distribution over time in (Figures 6D, 7D, 8D), it is additionally plotted on a logarithmic color scale in (Figures 6E, 7E, 8E). The corresponding development of the gain over time is plotted in (Figures 6F, 7F, 8F), and the bias in (Figures 6G, 7G, 8G). The correlation of the maximum DNF output *y*(*t*) with the corresponding input *z*(*t*) of the DNF is shown in (Figures 6H, 7H, 8H), computed for a sliding time window located at time step *t*:

(17) $$\begin{array}{l} corr(t)\;=\frac{\sum_{i=t-l}^{t}\left(z(i)-\bar{z})(y(i)-\bar{y}\right)}{\sqrt{\sum_{i=t-l}^{t}{\left(z(i)-\bar{z}\right)}^{2}{\sum_{i=t-l}^{t}\left(y(i)-\bar{y}\right)}^{2}}}, \end{array}$$

(18) $$\begin{array}{l} \bar{z}=\frac{1}{l}\overset{t}{\underset{i=t-l}{\sum }}\;z\left(i\right). \end{array}$$

The mean of the output ȳ is computed analogous to $\bar{z}$ (13). The length of the time window *l* is set to 5 min, just as for the computation of the sliding output distribution plots mentioned above. In the following, the results of the evaluation for different manipulations of the input statistics are presented.

#### 4.2.1. Low amplitude

In the first of this set of experiments, the input is down-scaled in its amplitude from a range of [0, 6] to [0, 1]. The results are shown in Figure 6.

The DNF is initialized with a bias (i.e., resting level) of −5 and a gain of 1 and has a recurrent interaction kernel which is kept constant for all experiments in this paper. The parameter adaption by IP results in the DNF output distribution shown in Figure 6B for the original input. At the 20th min the input is down-scaled by the factor of 6, which is too low to initiate DNF output activity. However, the gain and bias are adapted such that the DNF output distribution is restored within 10 min in the experiments. In particular, mainly the gain is adapted, the bias remains in its regime, which is expected as the input variance is manipulated. In Figures 6D,E this drop and the recovery in the output activity is visible in the histogram “gap” around the 25th min. The absence of DNF output is partly obscured, as the histogram is computed within a 5 min time window and the IP parameter adaption is completed within a similar time frame as visible in (Figures 6F,G).

While the output distribution is mostly restored by IP for a down-scaled input signal, the massive drop in the input-output correlation in Figure 6H indicates an additional aspect of the adaption with IP. The input signal *S*(*x, t*) is scaled by the gain *a* together with the recurrent interaction kernel ω(*x*−*x*′). As the gain is increased in order to compensate the decrease in the input signal intensity, the recurrent interaction is also increased. Thus, in this case there is a shift in the relative contributions of input and recurrent “feedback” to the current activity state *u*(*x, t*) of the DNF. The increased relative contribution of the recurrent component increases the stabilization of the DNF output and thus, reduces the input output correlation. This effect is also visible when comparing the final output distribution for the down-scaled signal in (Figure 6C) with the output distribution in (Figure 6B) for the original input signal. In Figure 6C there is an increase in the “high” output states near one and a decrease in the “medium” output activity.

#### 4.2.2. High amplitude

The second experiment with respect to varying the input statistics is analogous to the previous, except that the input is now scaled-up. After the initial parameter convergence to the original input signal, the input is scaled to [0, 36] at the 20th min. The re-adaptation of the IP parameters is then analyzed in Figure 7.

After the up-scaling of the input, the DNF output is driven into saturation for the majority of all inputs. This is reflected in Figures 7D,F, where a change in the output distribution is visible. Like in the previous experiment, the effect is partly obscured by the temporal integration within a 5 min time window in order to compute the histogram. As a consequence to the input up-scaling, the gain is lowered (Figure 7F) for an appropriate re-scaling of the input signal. Similar as in the previous case of a lowered input amplitude, the bias remains stable. The final output distribution in (Figure 7C) and at the 50th min in (Figures 7D,E) shows that the parameter adaptation by IP is capable to retain the desired target output distribution.

However, just as in the previous experiment, the compensation of a re-scaled input signal with the gain parameter shifts the relative contributions of input and recurrent interactions, in this case toward a higher contribution of the input signal. As the gain parameter is decreased, the recurrent interactions are weakened in their contribution to the DNF activation. Thus output peaks are less stabilized with respect to input fluctuations. This is visible especially in the input-output correlation in Figure 7H. Here the correlation reaches one, implying a strongly input driven system output. This decrease of recurrent interactions is also visible when comparing the output distributions before (Figure 7B) and after (Figure 7C) the input up-scaling. While for the original input the IP parameters lead to a suppression of intermediate outputs, these are more prevalent after the input down-scaling.

#### 4.2.3. High offset

In the last experiment, the input signal *S*(*x, t*) is shifted in its mean by 12, thus from the range [0, 6] to [−12, −6]. In contrast to the previous two experiments, in which the input signal was scaled, this experiment requires an adequate adaptation of the bias only in order to compensate the input shift. This experiment is further utilized to illustrate the impact of the natural gradient in the gradient descent. For this, the experiment is carried out twice: first the IP adaptation with the natural gradient (NG) will be described as before and then the case of adapting IP without the NG is compared.

In the left column in Figure 8 the case of the adaption with NG is illustrated, analogous to the previous experiments. After the 20th min the input is shifted, which leads to decreased output activity in the output histograms (Figures 8D1,E1). As visible in Figure 8G, the bias is adapted such that it compensates the shifted input signal. Although, the gain is initially modified, it converges back to the previous value, which will be discussed in a subsequent paragraph. When comparing the DNF output distributions in (Figures 8B1,C1), no difference is noticeable. This also holds for the output histograms (Figures 8D1,E1), which look the same at the 50th min as before the input shift at the 20th min.

In contrast to the previous experiments, the gain is ultimately not adapted, such that the relative contributions from the input signal and recurrent interactions remain the same. The input manipulation can be fully compensated by the additive bias. Therefore, the input-output correlation in (Figure 8H) also converges back to the previous value.

The decrease of the gain for inputs with high bias (i.e., shifts) is a “input variance overestimation” problem of the IP algorithm (Neumann et al., 2013). The input variance, i.e., the deviation of the input signal from zero, can be reduced by lowering the gain, thereby reducing the error of the output distribution with respect to the target exponential distribution. However, this is only a short term solution as for an increasing bias the optimal gain returns to the former value. The standard gradient descent of IP learning therefore drastically lowers the gain in order to increase it again when the bias has been adapted such that the input mean is compensated, visible in the orange graph in Figure 8F. In this case the computation of the natural gradient, i.e., the transformation of the gradient from the Euclidean space into the Riemannian space prevents the reduction of the gain to nearly zero and only leads to a slight input variance overestimation, visible in the de- and increase of the gain around the 25th min, shown in the blue graph in (Figure 8F). At this point in time the bias reaches a regime in which the input leads DNF output and the gain starts to converge back to the previous value. Thus, in this experiment the impact of the change of the gradient metric on the gradient direction is directly visible, as the adaptation of the gain in a “wrong” direction is reduced, compared to the adaptation without NG in (Figure 8F). Although, the two learning algorithms have the same learning rate of η = 0.001, the learning with NG is much faster. This is in particular visible when comparing the parameter adaptations in Figures 8F,G, but also when looking at the output histograms in Figures 8D2,E2. Note, that in the output distribution plotted in Figure 8C2 the parameter adaptation by IP has not converged yet. Altogether, the use of the NG leads to a significantly faster convergence with less fluctuations in the parameter adaptation.

## 5. Discussion

In this paper, the adaptation of dynamic neural fields by intrinsic plasticity is proposed, analogous to IP in single neuron models. The core idea behind our approach is, first, to define scalar measures of the input and output of the whole DNF. Here, the maximum output and the input at the corresponding location on the feature dimension are chosen. Second, a target distribution of the DNF output measure is defined, which determines the statistics of the output. Since we selected the maximum output as the output measure, the target distribution in our case characterizes the distribution of “peak,” i.e., detection, vs. “no peak,” i.e., non-detection, states. In this paper, the exponential distribution is chosen, analogous to the conventional IP learning in single neurons. However, the proposed approach is not limited to the exponential distribution, other target distributions as e.g., the Gaussian may be used. The choice of this target distribution for IP will shape the overall dynamics of the DNF. If the DNF output should spend more time in the activated state the Kumaraswamy's double bounded distribution parameterized with a + b = 1.0 could be an interesting candidate (Kumaraswamy, 1980).

These design choices enable to derive learning rules for IP, which adapt the bias (i.e., resting level) and the gain in order to approximate the target distribution of the DNF's output. For an appropriate kernel parametrization, IP ensures a highly input sensitive operating regime for the DNF dynamics, defined by the hyper-parameters of the target distribution. Therefore, only the DNF recurrent interaction kernel parameters remain to be tuned manually. This autonomous adaptation of the DNF resting level and gain is in particular relevant for architectures in which DNFs receive inputs with unknown distributions, but for which the desired output distribution is known, as in our example in the introduction, where 20% of the most circular contacts should be detected as being “flat surfaces,” i.e., should produce a suprathreshold activity peak. Furthermore, a DNF with IP is capable to compensate moderate changes in the input amplitude (i.e., variance) and mean—however, at the cost of a shift in the relative contributions of input and recurrent interactions to the DNF output. This shift in relative contributions is a clear limitation of the proposed approach when large changes in the variance of the input signal are expected, as revealed in the high- and low-amplitude experiments.

Adaptation in our model changes a *global* gain and bias for the entire neuronal population, modeled by the DNF, in contrast to independent adaptation of a local gain and bias for every neuron in the population. Formulation of IP for population codes in a local, single neuron based fashion is not straight-forward: In population encoding, the activity of a neural field encodes the confidence that the input feature has the value, to which the underlying neurons are tuned, i.e., the neuron has a local receptive field in the input space of the DNF. It is only for a neuron that encodes relevant feature values (i.e., the input regularly falls within the receptive field of the neuron) that an adaptation of intrinsic excitability makes sense. In order to realize an individual adaptation of gains and biases of single neurons, there needs to be an additional mechanism in place to adapt the receptive field position (i.e., the input weights) of each neuron, i.e., to tune the neuron to represent a new feature value. This would correspond to an adaptive feature resolution on the population level, with fovea-like effects for feature value regions with high probability. The originally proposed algorithm of the self organizing maps (SOM) would be an example of such a receptive field tuning of a DNF (Kohonen, 1982). The problem here is the strong dependence on stochastic, uncorrelated input required for training and maintaining the SOM, which renders the SOM algorithm inapplicable for highly correlated in time inputs. This motivates the tuning of global parameters for the entire population.

There is also a motivation from the biological perspective for the global adaptation based on IP. In addition to the plasticity of excitability in individual neurons and their dendritic structures, accumulating evidence exists of neuronal mechanisms that perform a multiplicative normalization of entire populations of neurons (Carandini and Heeger, 2012). The existence of global, network-wide activity regulation in addition to single neuron and synaptic adaptations is also proposed in Slomowitz et al. (2015) based on recordings of cultured hippocampal networks. In particular, a coupled gain and bias adaptation among neuronal populations has been proposed as an explanation for results from large-scale recordings in the primary visual cortex (V1) (Lin et al., 2015). These biological findings additionally motivate the proposed implementation of IP in DNFs in this paper, based on a coupled gain and bias for an entire population of neurons.

This paper also shows the limits of the adaptation by IP, in particular when the amplitude of the input signal (i.e., variance) is subject to strong changes. If the input amplitude declines too much, the increasing gain will eventually reach a regime, where the recurrent feedback self-stabilizes the DNF output—independent of the input. In this case the adaptation will lower the gain and bias again, leading to an on-off oscillation of the DNF output. This corresponds to the results by Marković and Gros (2010, 2012), where the authors demonstrate that IP leads to the destruction of the attractor stability, leading to oscillatory, and bursting behavior of recurrent neural networks with no- or very small inputs.

Despite these limitations for strong changes in the input distribution, this paper shows that the adaptation of DNFs with IP is feasible and can be used in applications, in which a DNF architecture is driven by sensory inputs whose statistics is not known in advance or may change over time. Examples of such applications could be, e.g., color vision at varying illumination, or auditory perception with different levels of background noise. The benefit of this adaptation is that it simplifies tuning and allows application of DNFs to inputs whose distribution is only roughly known (e.g., in terms of the min and max values) while the desired distribution of DNF output can be specified in advance. In such cases, the definition of a recurrent interaction kernel and a desired output distribution with its hyper-parameter(s) drive self-adaptation of the DNF.

## Author contributions

CS was the main driving force behind this work, it is part of his doctoral thesis; GS was providing guidance of the theory in the paper that concerns DNFs; FW was providing guidance in parts that concern unsupervised learning and IP; YS was the day-to-day supervisor of CS and provided support in both theory and implementation for the robotic application; all authors contributed to writing and revising the manuscript.

### Conflict of interest statement

The authors declare that the research was conducted in the absence of any commercial or financial relationships that could be construed as a potential conflict of interest.
